# Editorial: The mechanism of plant-derived polysaccharides regulating the obesity and metabolic diseases in humans

**DOI:** 10.3389/fnut.2022.988653

**Published:** 2022-11-04

**Authors:** Guiguo Zhang, Yunkyoung Lee, Yang Liu, Yanling Hao

**Affiliations:** ^1^Department of Animal Nutrition, China-Korea Joint R&D Center on Plant-Derived Functional Polysaccharide, Shandong Agricultural University, Taian, China; ^2^Department of Food Science and Nutrition, Korea-China Joint R&D Center on Plant-Derived Functional Polysaccharide, Interdisciplinary Graduate Program in Advanced Convergence Technology & Science, Jeju National University, Jeju, South Korea; ^3^Guangdong Provincial Key Laboratory of Marine Biotechnology, Department of Biology, College of Science, Shantou University, Shantou, China; ^4^Department of Nutrition and Health, China Agricultural University, Beijing, China

**Keywords:** plant-derived polysaccharides, obesity, metabolic disease, microbiome, metabolome

Obesity and other metabolic diseases, such as non-alcoholic fatty liver disease and type 2 diabetes, have become some of the primary threats to human health, and the occurrence of these diseases generally means the disturbance of glucose and lipid metabolisms in the body. Emerging studies have shown that mechanisms of metabolic diseases can be directly attributed to disorders of tissue metabolism (1, 2) and the disruption of intestinal microbiota homeostasis (3, 4). Plant-derived polysaccharide (PS) is one of the most effective prebiotics for improving the abundance of beneficial gut microbes (5), which exert a variety of health-promoting effects (6), including antioxidant, anti-inflammation (Cui et al.), immunomodulation, antiviral, anti-diabetes (7), and anti-obesity effects (6). It has been documented that PSs from different plants have differentiated structural properties and varied capacities to interact with specific cells (8) and/or shape unique gut microbial communities (9), which furthermore impact the physiological and pathological metabolic processes of the host (10, 11). Thus, clarifying the molecular structure, bioactivities, and mechanisms by which PS modulates the occurrence of obesity and metabolic disease is of great importance for developing and applying natural PS as a pharmaceutical and functional food.

A total of five papers have been published in this special issue, which present the latest research advances in the field investigating the structural characteristics and bioactivities of plant-derived PSs involved in regulating metabolic diseases. One of the papers focused on the structural characteristics and bioactivities of *Laminaria japonica*-derived PS (LJPS). A similar study explored the effects and mechanisms of dietary fibers (PS) from medicinal *Dendrobiums* for diabetes management. A further study revealed that dietary PS (whole grain Qingke) can attenuate high-fat diet induced obesity by modulating the gut microbiome and metabolome. Finally, two articles addressed the feasibility of microbial and gene therapeutics based on the consumption of dietary fibers/plant-derived PSs in treating metabolic syndrome (MetS). Thus, the five articles documented innovative findings in deciphering the structure and bioactivities of various plant-derived PSs from different perspectives.

LJPS were documented to have a variety of health-beneficial biofunctions, including anti-oxidation, anti-inflammatory, and lipid-lowering biofunctions, while molecular structure characteristics, especially spatial conformation, remained unclear. Cui et al. revealed that LJPS was a multi-branched, long-chain macromolecule, and appeared in a denser cross-linking network with highly branched and helix domains in terms of morphology. Additionally, LJPS had no toxicity in the macrophage cells of mice and exhibited biphasic immuno-modulating capacity.

It has been documented that the biofunction of medicinal plants used in traditional medicine was attributed to their rich PS content (12). Li M. et al. observed that PS and bibenzyl are the major active compounds in *Dendrobiums* in managing diabetic-related symptoms *via* lowering blood glucose levels and reversing chronic inflammation of type 2 diabetic mellitus (T2DM). *Dendrobiums* PS protected pancreatic β-cell dysfunction and insulin resistance in the liver, and up-regulated the abundance of short-chain fatty acid to stimulate GLP-1 secretion through gut microbiota. Similarly, bibenzyls also exerted the capacity to prevent chronic inflammation in cellular studies.

The beneficial effects of fiber-rich dietary food on improving the systematic health of humans have been related to alteration of the gut microbial community (2). Li X. et al. elucidated that whole grain Qingke (WGQK) had an anti-obesity effect in a diet-induced obesity model in mice by modulating the gut microbiota and their metabolome, primarily shifting the host amino acid/lipid metabolism. Metabolic syndrome (MetS), accompanied by significant intestinal dysbiosis, is a major health burden to human society. Pan et al. discovered that microbial therapy is an efficient strategy to treat and remit MetS, notably improving the condition of fasting blood glucose (FBG), total cholesterol (TC), triacylglycerol (TG), high-density lipoprotein cholesterol (HDL-C), low-density lipoprotein cholesterol (LDL-C), waist circumference (WC), body mass index (BMI), homeostatic model assessment of insulin resistance (HOMA-IR), and diastolic blood pressure (DBP) of patients. This provided an innovative insight in treating MetS by supplementing the diet with dietary PSs/fiber to modulate the targeted gut microbes and their metabolites. Similarly, Gu et al. summarized the progress of new gene therapy in treating MetS. It has been documented that MetS onset is closely related to impaired lipid metabolism, and regulating the lipid metabolic genes would provide an advanced perspective in developing MetS therapeutics. Additionally, recent studies revealed vital functions of nuclear receptor (NR) retinoic acid receptor-related orphan receptors (RORs), including RORα and RORγ, in gene regulation in lipid metabolism in MetS. These results also provided a theoretical support for exploring the molecular mechanisms and new therapeutic strategies regarding MetS.

It should be pointed out that the five articles published on this specific topic were conducted mainly by using mice, or cells from mouse tissue, to allow the model to explore the biological activities and mechanisms of plant-derived PS in managing metabolic diseases such as obesity, type 2 diabetes, and non-alcoholic fatty liver disease. These findings provided new perspectives and methods for the application of plant-derived PS to prevent and/or treat metabolic diseases in humans, but relevant clinical trials are necessary to ascertain the effective and appropriate dose before applying as therapeutic agents for humans.

In conclusion, plant-derived PS is one of the most functional ingredients regarding its wide variety of bioactivities and shows great potential especially regarding the positive therapeutic effects on metabolic diseases including obesity, non-alcoholic fatty liver, and diabetes, primarily by specifically manipulating gut microbiota and their metabolites. It is worth noting that the molecular structural characteristics and chain spatial configuration of plant-derived PS are responsible for their bioactivities. Therefore, it is pivotal to decipher the molecular properties and the structural-function relationship of PSs, which would provide the scientific reference for applying natural PSs to the pharmaceutical and functional food industries.

## Author contributions

The idea and concept of this Research Topic came from a discussion among the guest editors. GZ and YLee finalized the writing of this editorial. YH and YLiu gave some valuable suggestions. All authors contributed to the writing process of the editorial and approved the final version of it.

## Funding

This work was supported by the National Key R&D Program of China-Korea cooperative project (2019YFE0107700 and NRF-2019K1A3A1A20081146), the National Research Foundation Grant of Korea (2017R1D1A3B03031665 and 2020R1A2C2004144), the Forage Industrial Innovation Team Project (SDAIT-23-05), Shandong Province Technology Innovation Guidance Plan (2019YFE0107700), the Excellent Seed Project (2019LZGC012) of China, the Educational Commission of Guangdong Province of China (2021ZDZX2051), and Li Ka Shing Foundation Cross-Disciplinary Research Grant (2020LKSFG02E).

## Conflict of interest

The authors declare that the research was conducted in the absence of any commercial or financial relationships that could be construed as a potential conflict of interest.

## Publisher's note

All claims expressed in this article are solely those of the authors and do not necessarily represent those of their affiliated organizations, or those of the publisher, the editors and the reviewers. Any product that may be evaluated in this article, or claim that may be made by its manufacturer, is not guaranteed or endorsed by the publisher.
